# Influence of malformation of right coronary artery originating from the left sinus in hemodynamic environment

**DOI:** 10.1186/s12938-020-00804-0

**Published:** 2020-07-29

**Authors:** Mengyang Cong, Xingming Xu, Jianfeng Qiu, Shun Dai, Chuanzhi Chen, Xiuqing Qian, Hongbin Zhang, Shengxue Qin, Huihui Zhao

**Affiliations:** 1 College of Mechanical and Electronic Engineering, Shandong University of Science and Technology, Qingdao, 266590 China; 2 Intelligent Equipment College, Shandong University of Science and Technology, Taian, 271016 China; 3 Department of Radiology, Shandong First Medical University & Shandong Academy of Medical Sciences, Taian, 271016 China; 4 Center for Medical Engineer Technology Research, Shandong First Medical University & Shandong Academy of Medical Sciences, Taian, 271016 China; 5 Department of Radiology, Shanghai Tong Ren Hospital, Shanghai Jiaotong University School of Medicine, Shanghai, 200120 China; 6 Department of Radiology, East Hospital, Tongji University School of Medicine, Shanghai, 200120 China; 7 Department of Biomedical engineering, Capital Medical University, Beijing, 10060 China

**Keywords:** Computational fluid dynamics, Anomalous right coronary artery, Volumetric flow, Pressure, Wall shear stress, Statistical analysis

## Abstract

**Background:**

The anomalous origin of the right coronary artery (RCA) from the left coronary artery sinus (AORL) is one of the abnormal origins of the coronary arteries. Most of these issues rarely have any effects on human health, but some individuals may exhibit symptoms, such as myocardial ischemia or even sudden death. Recently, researchers have investigated the AORL through clinical cases, but studies based on computational fluid dynamics (CFD) have rarely been reported. In this study, the hemodynamic changes between the normal origin of the RCA and the AORL are compared based on numerical simulation results.

**Methods:**

Realistic three-dimensional (3D) models of the 16 normal right coronary arteries and 26 abnormal origins of the RCAs were constructed, respectively. The blood flow was numerically simulated using the ANSYS software. This study used a one-way fluid–solid coupling finite element model, wherein the blood is assumed to be an incompressible Newtonian fluid, and the vessel is assumed to be made of an isotropic linear elastic material.

**Results:**

The cross-sectional area differences between the inlet of the normal group and that of the abnormal group were significant (*P *< 0.0001). Moreover, there were significant differences in the volumetric flow (*P *= 0.0001) and pressure (*P *= 0.0002). Positive correlation exists for the ratio of the cross-sectional area of the RCA to the inlet area of the ascending aorta (AAO), and the ratio of the inlet volumetric flow of the RCA to the volumetric flow of the AAO, in the normal (*P *= 0.0001, *r* = 0.8178) and abnormal (*P *= 0.0033, *r* = 0.6107) groups.

**Conclusion:**

This study demonstrates that the cross-sectional area of the AORL inlet may cause ischemia symptoms. The results obtained by this study may contribute to the further understanding of the clinical symptoms of the AORL based on the hemodynamics.

## Background

The coronary artery originating from the root of the aorta is divided into the left coronary artery (LCA) and the right coronary artery (RCA). The RCA originating from the right aortic sinus of the heart supplies blood to the superior and inferior ventricles at the right side of the heart. The anomalous origin of the coronary artery (AAOCA) is a common congenital coronary artery anomaly [1]. Although some children are born with AAOCA, the condition may not be diagnosed during coronary angiography until the individual grows to an adolescence or adulthood [2–4]. There are four main types of AAOCA: (a) absence of left main artery; (b) abnormal origin of the coronary artery from the improper sinus; (c) anomalous coronary ostium outside of Valsalva’s sinus; (d) a single coronary artery [1]. Although most coronary artery anomalies only have slight effects on individuals, some young individuals, who are prone to sudden cardiac death (SCD), typically exhibit the two common abnormality characteristics: RCA and abnormal LCA [4]. This study focused on the hemodynamics of the RCA originating from the left coronary sinus.

In patients undergoing coronary angiography the incidence of the anomalous origin of the RCA from the left sinus (AORL) is 0.92% [5]. Compared with the origin of the LCA, the AORL is responsible for a lower rate of sudden death. The incidence of typical angina and myocardial infarction in patients with low interarterial course in the AORL is significantly lower than that for patients with high interarterial course [6]. However, although the incidence of AORL-caused cardiovascular disease is relatively low, the consequences are often fatal.

Studies have reported that the AORL can cause myocardial ischemia, angina pectoris, myocardial infarction, and SCD [7–9]. Moreover, it has been reported that an acute AORL angle may cause cardiovascular disease during exercise [10]. The most proximal part of the artery has an oblique intramural course with a slit-like ostium, which may cause ischemia [11]. The mechanical compression of the RCA caused by the dilation of the pulmonary artery and aorta may result in ischemia during intense exercise [12, 13].

Initially, one of the causal mechanisms of SCD was thought to be associated with the slit-like or flap-like closure of the orifice [14, 15]. The data reported from a large series of clinical studies based on intravascular ultrasonography (IVUS), which were conducted on 63 adult patients with AORL, demonstrate that the severity of stenosis is related to the occurrence of ischemic symptoms (syncope, angina, or dyspnea, in addition to SCD) [16]. In this study, the cross-sectional area at the RCA inlet of the abnormal group was much smaller than that of the normal group. One of the AORL features is a slit-like orifice [17], Therefore, the cross-sectional area is small, and this may cause the functional stenosis of the AORL, ischemia, and malignant ventricular arrhythmias [15].

In this study, the hemodynamics of the AORL were investigated using computational fluid dynamics (CFD). As a branch of hydrodynamics, the CFD method is increasingly being used in biomedical engineering, and many bioengineers have used CFD to investigate complex cardiovascular diseases, such as coronary artery stenosis [18–23]. We hypothesized that the hemodynamics of the RCA of normal origin are different to those of the AORL. A total of 42 realistic geometric AORL models were constructed based on computed tomography angiography (CTA) scan images, and then the hemodynamics were simulated using a numerical method. The main objective of this study was to investigate the effects of the AORL on hemodynamics, and the findings have theoretical significance for the clinical evaluation of ischemic symptoms.

## Results

Before ANSYS analysis, the material parameters were set as follows: the blood density and viscosity were set to be constant at 1060 kg/m^3^ and 3.5 × 10^−3^ Pa·s, respectively [24, 25]. The blood flow was set to no heat exchange and laminar flow. The material of the fluid part was set to the blood flow parameters. The vessel wall density was 1150 kg/m^3^, the Young’s modulus was 5 MPa, and the Poisson’s ratio was 0.45 [25].

**Fig. 1:**
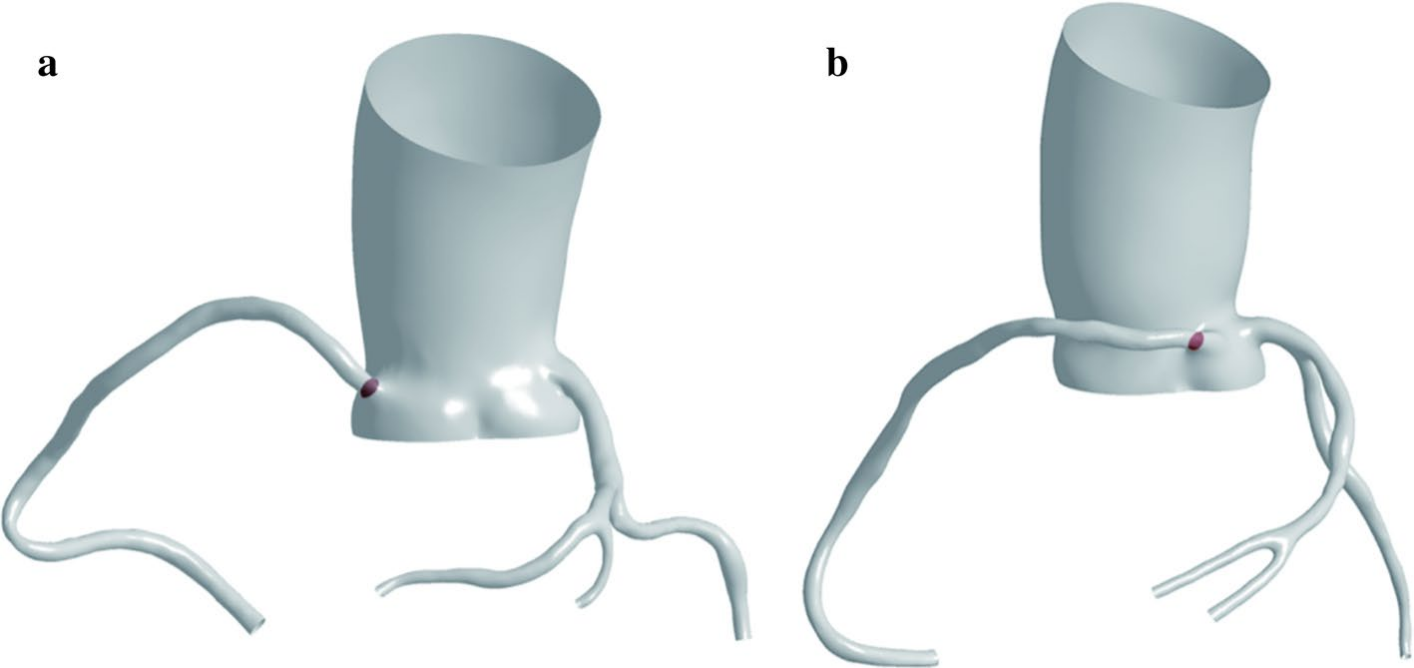
Position of RCA inlet velocity: **a** normal origin of RCA; **b** anomalous origin of RCA from left coronary sinus of Valsalva. Two examples are used to indicate the entrance location

**Fig. 2:**
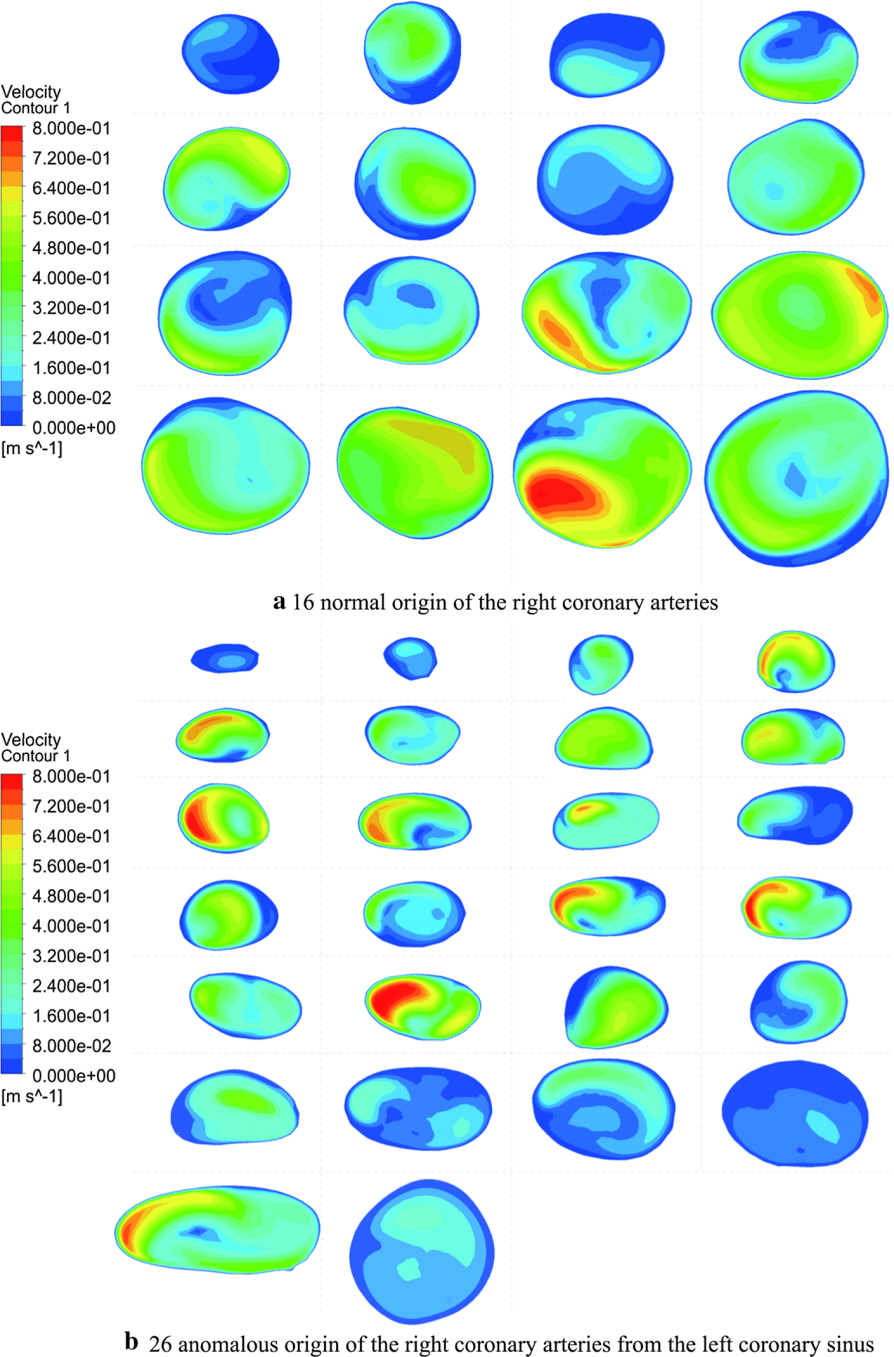
Flow rate of entrance section of RCA. Owing to individual differences, the area and shape of the entrance section of each model are different. The flow of the inlet section for the normal and anomalous groups was separately read by two contours

**Fig. 3:**
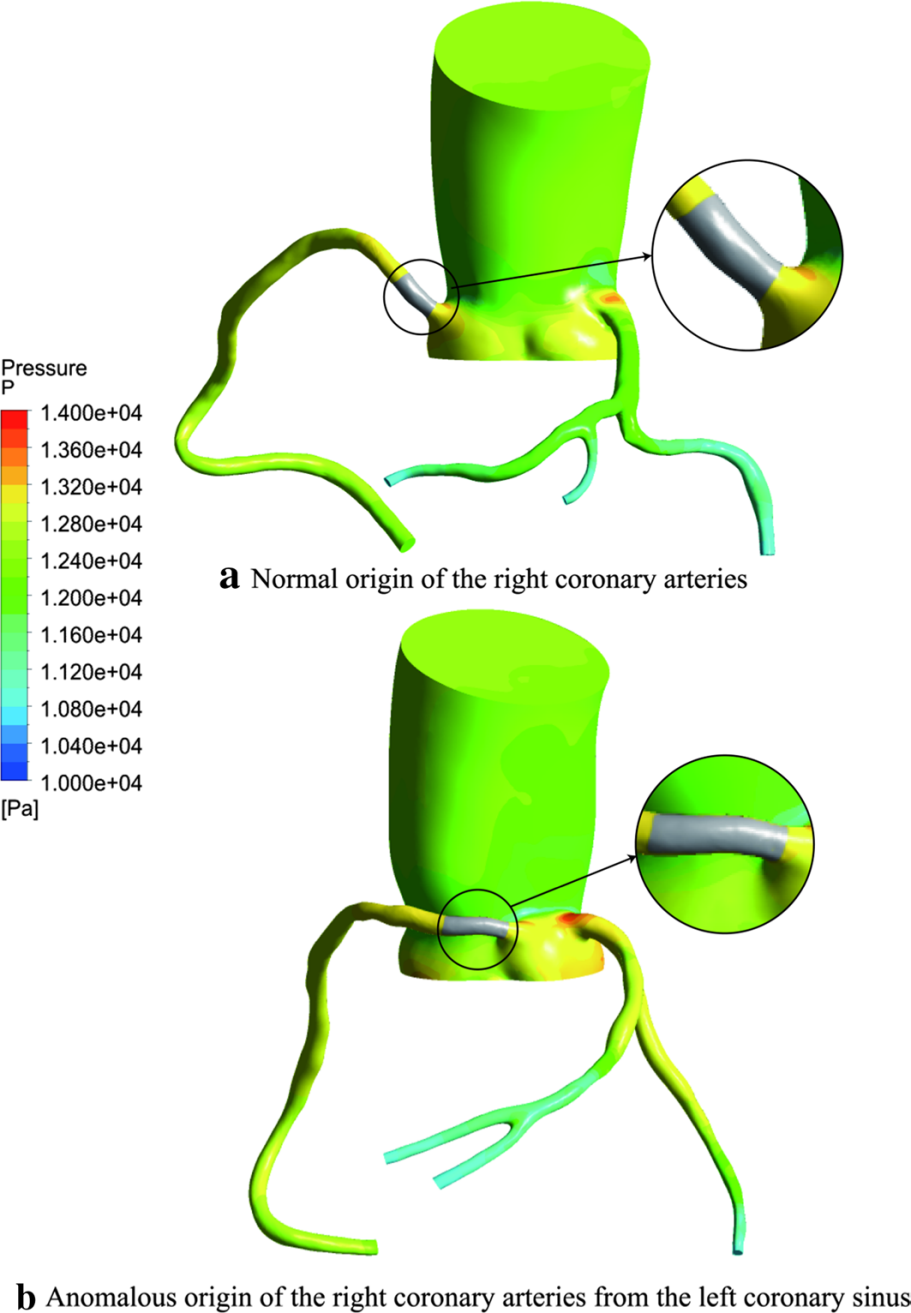
Location selected using five-point sampling method. The gray area is the value area of the model; the value area is enlarged three times to show the details

The red section is the cross-sectional area of the RCA inlet (Fig. 1). The flow rate of the AORL was smaller than that of the normal coronary artery (Fig. 2). In each model, the average pressure and wall shear stress (WSS) at the corner of the RCA were calculated using five-spot sampling. The gray part is the range of the five-point sampling method and the length is approximately 10–15 mm (Fig. 3).

According to the hemodynamic parameters of the abnormal group, one case of RCA stenosis and three cases of RCA originating from the ascending aorta were removed, and the data of the remaining cases were statistically analyzed. The ratio of the cross-sectional area of the RCA to the inlet area of the ascending aorta (AAO) is defined as follows:

$$S_{\% } \, = \,S_{\text{RCA}} /S_{\text{AAO}} .$$

The ratio of the inlet volumetric flow of the RCA to the volumetric flow of the AAO is defined as follows:

$$Q_{\% } \, = \,Q_{\text{RCA}} /Q_{\text{AAO}} .$$

**Fig. 4:**
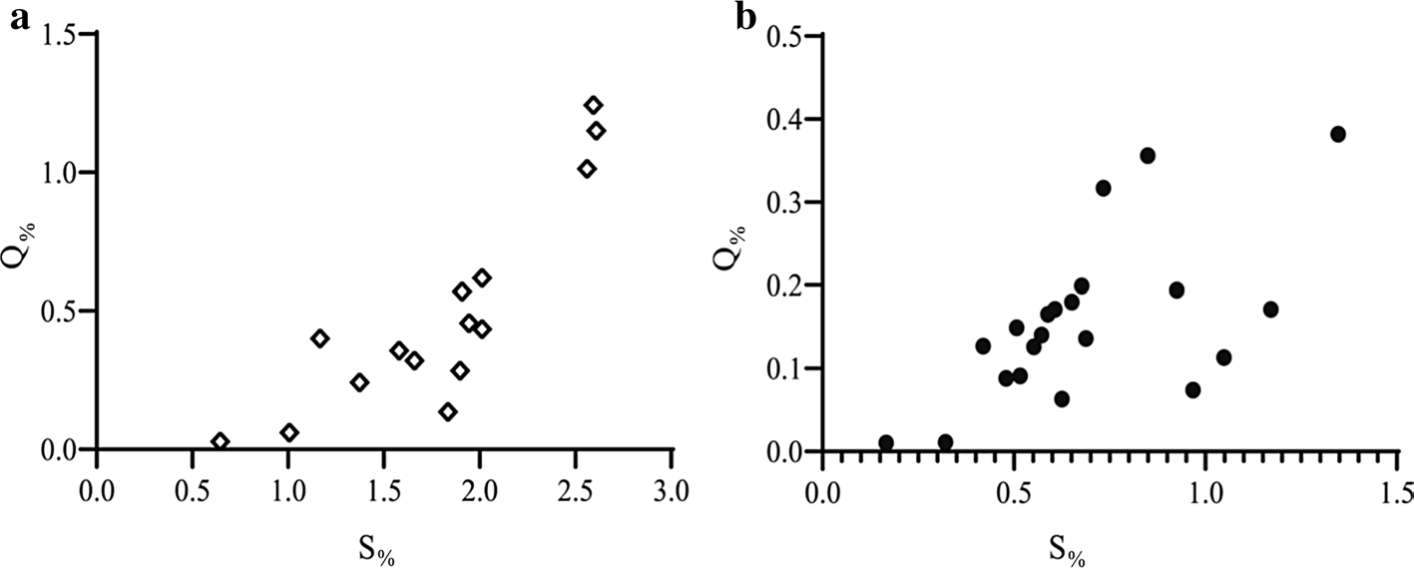
**a** Normal origin of RCA; **b** anomalous RCA from LCA sinus; *S* _%_ is the ratio of the cross-sectional area of the RCA to the inlet area of the ascending aorta; *Q* _%_ is the ratio of the inlet volumetric flow of the RCA to the volumetric flow of the ascending aorta

**Fig. 5:**
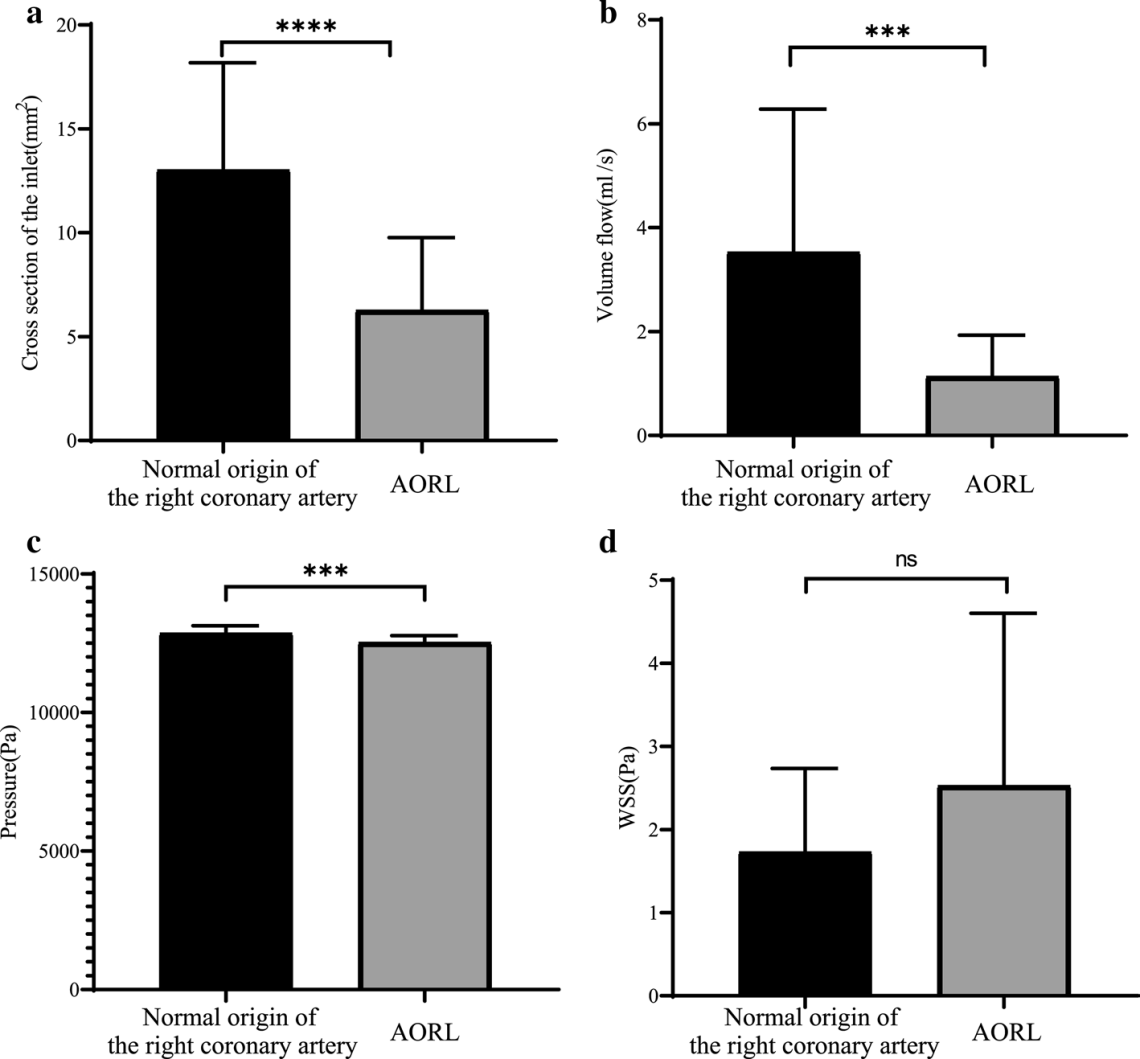
Comparison of inlet cross-sectional area, volume flow, pressure, and WSS between abnormal group and normal group: ****P < 0.0001; ***P = 0.0001; ns *P* > 0.05

In the abnormal group, there were obvious positive correlations between *S* _%_ and *Q* _%_ (*P* = 0.0033, *r* = 0.6107) (Fig. 4a). The *S* _%_ of the normal group exhibits great positive correlation with *Q* _%_ (*P* = 0.0001, *r* = 0.8178) (Fig. 4b). There were significant differences in the cross-sectional area of the inlet (*P *< 0.0001) (Fig. 5a). The average cross-sectional area of the normal group (13.02 ± 1.333 mm^2^) was twice that of the abnormal group (6.268 ± 1.333 mm^2^). The volumetric flow rate (*P *= 0.0001) and pressure (*P *= 0.0002) differences between the normal group and the abnormal group are obvious (Fig. 5b, c). The average volume flow in the abnormal group (1.135 ± 0.5714 mL/s) was approximately one-third of that in the normal group (3.529 ± 0.5714 mL/s). In terms of specific values, there is no significant average pressure difference between the normal group (12,861 ± 80.47 Pa) and the abnormal group (12,532 ± 80.47 Pa). No significant difference was observed for WSS (*P *> 0.05) (Fig. 5d). The average WSS of the normal group (1.730 ± 0.5561 Pa) is approximately the same as that of the abnormal group (2.529 ± 0.5561 Pa).

## Discussion

Most studies have investigated the AORL through clinical cases and anatomical studies, but few studies have carried out quantitative analyses on hemodynamics to address this problem [26–30]. To investigate the difference between the hemodynamics of the normal RCA and the AORL, we simulated the human coronary artery hemodynamics (the model includes LCAs and RCAs, and a partial aorta) based on multi-slice CTA scan images. The effects of the slit-like closure of the AORL on the hemodynamic parameters and clinical symptoms were investigated by comparing the inlet volumetric flow, pressure, and WSS data of the RCA.

The correlation between the area ratio and the flow ratio was analyzed to eliminate the individual vascular differences in each patient. This study demonstrated that *Q* _%_ decreased with *S* _%_ in both the normal and abnormal groups. Additionally, it was found that the *S* _%_ and *Q* _%_ of the abnormal group were smaller than those of the normal group. One reason for this is that, even though the velocity increased owing to the decrease of the cross-sectional area of the AORL inlet, the volumetric flow into the RCA in the abnormal group eventually became three times smaller than that in the normal group. Another reason for this may be that blood is forced into the LCA owing to the close distance from the starting position of the LCA and RCA. In the abnormal group, the volumetric flow into the RCA was smaller. This leads to insufficient blood flow at the end of the RCA, which is unable to exchange blood with the heart in a timely manner, and this eventually leads to myocardial ischemia, such as angina [33].

Recently, by comparing the hemodynamic parameters of groups with and without stenosis, it was found that the coronary artery stenosis group had lower pressure [31]. Additionally, it was found that the cross-sectional area of the abnormal group inlet was smaller than that of the normal group, and the pressure at the corner of the abnormal group was smaller than that of the normal group. The fluid energy comprises the potential energy, pressure energy, and kinetic energy. The decrease in the cross-sectional area of the abnormal group leads to velocity increase. According to the conservation of energy law, the pressure is reduced, which agrees with the results obtained by this study. If the RCA entrance is very narrow, the blood flow to the heart decreases, particularly when the heart is beating fast. Eventually, the decreased blood flow may lead to shortness of breath, angina, or other signs and symptoms of coronary artery disease [32, 33].

Pathological studies on coronary artery disease have reported that atherosclerosis is the main cause of myocardial ischemia in adults [34]. The WSS plays an important role in the development and rupture of atherosclerosis [35]. This study found that the arterial WSS reduction is positively correlated with the decrease of the artery wall’s elasticity, and that the consideration of the WSS is effective for the early atherosclerosis diagnosis [36, 37]. This study found that there is no significant difference between the mean WSS value of the abnormal group and normal group. We believe that a slit-like or flap-like closure can affect the WSS of the RCA in the AORL. As revealed by the results, the WSS of both the normal and abnormal group is larger than the range of the low-level WSS [38]. Therefore, the WSS comparison reveals that AORL patients have the same risk for developing atherosclerosis as normal patients, and the risk of atherosclerosis does not increase [38, 39].

This study has various limitations. Notably, the results reveal that the WSS is more accurate when using the heart arterial cycle as the inlet flow [40]. In future work, additional statistical analyses will be performed using the cardiac arterial cycle for bidirectional fluid–solid coupling.

## Conclusion

The main objective of this study was to analyze the AORL hemodynamics using CFD. The results reveal that the AORL led to a decrease in the volumetric flow and pressure at the RCA, but there were no differences in the WSS. Hence, it is concluded that the cross-sectional area of the AORL inlet, which changes the hemodynamic environment, can cause ischemia. This study focused on the cross-sectional inlet area of the RCA of the AORL, and clarified, from a hemodynamic perspective, that a slit-like orifice does not cause changes in the blood flow characteristics. Finally, the results of this study can be useful to clinicians as numerical guidelines for the diagnosis of ischemic symptoms in the AORL.

## Materials and methods

### Image collection

A total of 16 normal coronary artery and 26 abnormal coronary artery CTA images were collected using a 128-slice Siemens (SOMATOM Definition Flash) dual-source computed tomography (CT) scanner (80 kV, 140 kV). The tube voltage was selected according to the body mass index (BMI), as follows: for BMI ≤ 18, tube voltage = 80 kV; for 18 < BMI ≤ 22, tube voltage = 100 kV; for 22 < BMI ≤ 27, tube voltage = 120 kV; for BMI > 27, tube voltage = 140 kV. The contrast injection rate was 5–5.5 mL/s, and its dose was 60–80 mL. Drugs for controlling the heart rate were not administered to the patients before scanning. Additionally, 0.5 mL of nitroglycerin was sublingually administered before scanning, and the patients were scanned after breath training, while in a calm state. The scan settings are listed in Table 1.

**Table 1:** Scan settings

Parameter	Value
Rotation speed	280 ms/r
Collimator	2 × 128 × 0.6 mm
Slice thickness	0.75 mm
Slice increment	0.50 mm
In plane resolution	512 × 512

### Artery models

**Fig. 6:**
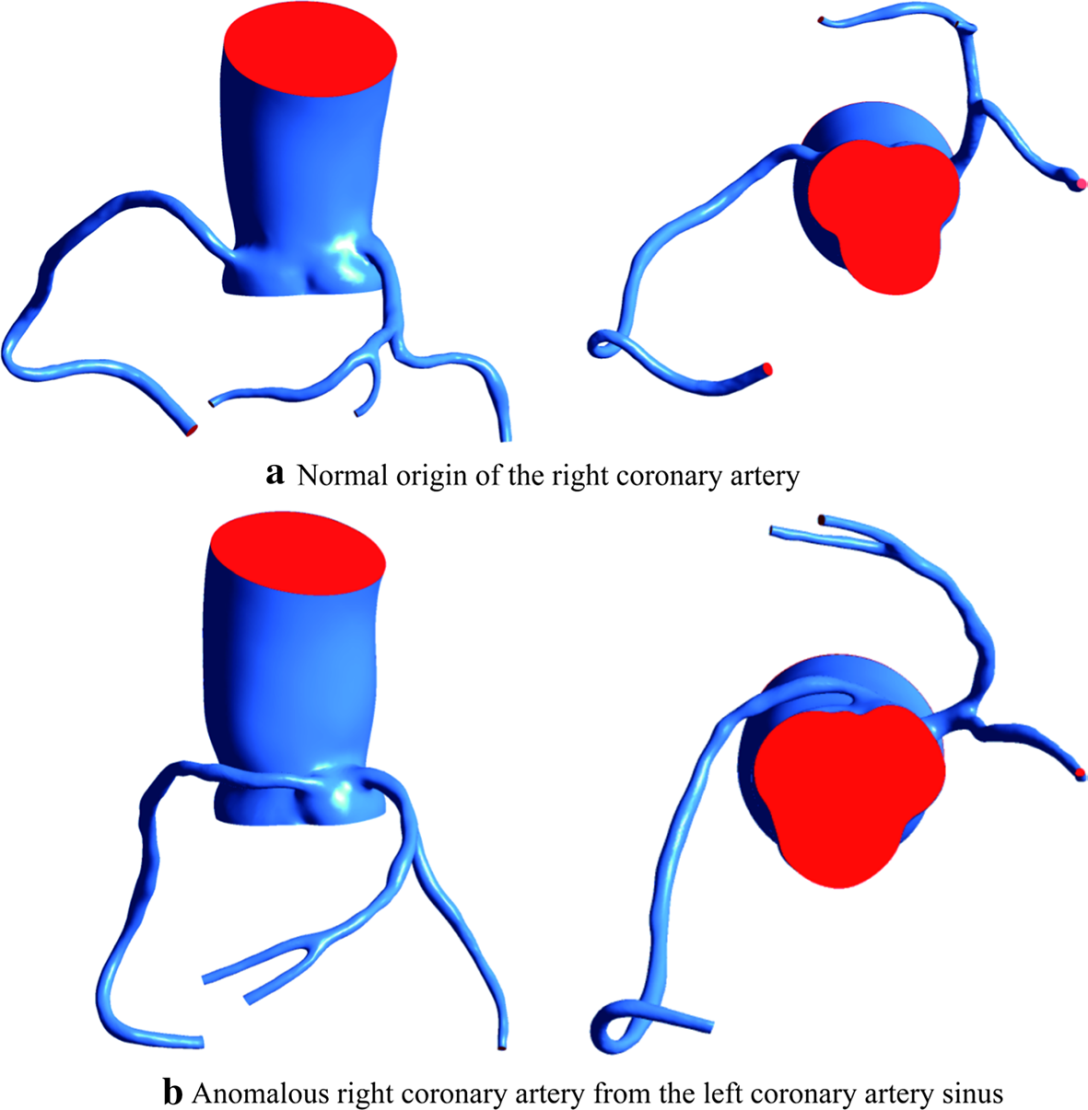
Front view and upward view of two coronary artery model examples

Realistic coronary arterial models were constructed based on samples collected by Mimics software (v. 9.0, Materialise, Ann Arbor, MI, USA). Geomagic Studio 2013 (3D Systems, Morrisville, NC, USA) was used to smooth the rough surfaces of the constructed models and subsequently generate solid surfaces. To simplify the numerical simulation, the small coronary branches of the coronary arterial models were removed. The main branch of the RCA was retained, and the left anterior descending coronary artery, left main coronary artery, and left circumflex coronary artery were retained for the LCA. The ascending aorta was cut off approximately 5.5 mm away from the coronary sinus, which is considered as the blood flow inlet. The surface of the reconstructed model was offset by 0.5 mm along the normal direction to obtain the vascular outer wall. The 3D CAD software Solidworks (Solidworks Corporation, Boston, MA, USA) was used to reconstruct the vessel wall of the model. The vessel wall was created by reconstructing the blood solid and offset solid. The blood model is shown in Fig. 6.

### Assumption and governing equations

The blood was considered as a homogenous and incompressible Newtonian fluid [20], and assumed to be isothermal. The blood flow is described by the 3D incompressible Navier–Stokes equations and continuity equation [41], as follows:

1 $$\frac{\partial u}{\partial t} + \left( {u \cdot \nabla } \right)u = \frac{1}{\rho }\nabla \cdot \sigma ,$$

2 $$\nabla \cdot u = \text{0,}$$

where *u* and *σ* represent the fluid velocity vector and stress tensor, respectively, and *ρ* is the density; *σ* is defined as follows:

3 $$\sigma = 2\eta \left( {\mathop \gamma \limits^{ \cdot } } \right)D + { - }pI,$$

where *η* and $\mathop \gamma \limits^{ \cdot }$ denote the blood viscosity and shear rate, respectively; *p* is the pressure; *D* is the rate of the deformation tensor, and is defined as follows:

4 $$D = \mu \left[ {\nabla u + \left( {\nabla u} \right)^{T} } \right],$$

where *μ* is the blood viscosity.

The vessel wall was considered as an isotropic and non-linear elastic material without infiltration. The equation governing the solid domain is expressed as follows:

5 $$\nabla \cdot \sigma_{s} = \rho_{s} \cdot \alpha_{s} ,$$

where *σ*_*s*_ is the stress tensor, *ρ*_*s*_ is the density, and *a*_*s*_ is the acceleration.

### Mesh generation

The meshes were generated using the ICEM software (ANSYS, Inc., Canonsburg, PA, USA). The geometric models were meshed using unstructured tetrahedral volume meshes. For the fluid portion of the model, the minimum and maximum mesh sizes were 0.06 mm and 1 mm, respectively, and five fine mesh layers with a height ratio of 1.2 were used. For the solid portion of the model, the mesh size was set to 0.5 mm.

### Boundary conditions

**Fig. 7:**
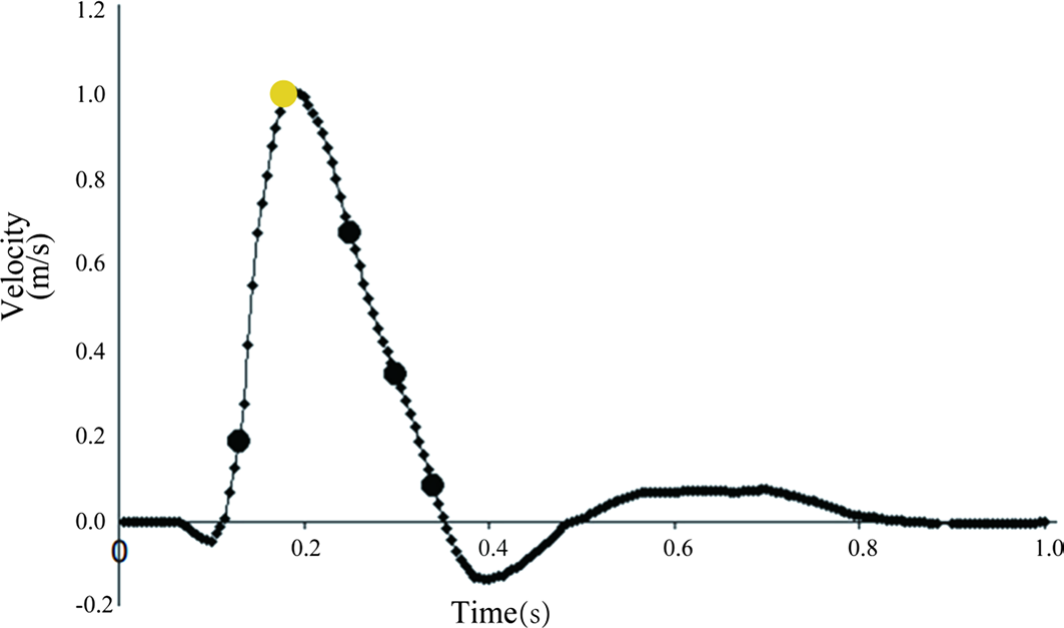
The exit velocity of the left ventricle is measured during a complete heart cycle. For stationary simulation, the entry boundary condition chosen was the maximum velocity. The velocity was 1.0 m/s

In this study, the inlet flow velocity was set as constant, which is a simple method for comparing the different effects of normal RCAs and abnormal RCAs on hemodynamics. The maximum exit velocity of the left ventricle was set as the entry boundary condition in the numerical simulation, as indicated by the yellow dot in Fig. 7 [42]. The mean arterial pressures of the aorta, LCA, and RCA were calculated according to the literature [43]. The outlet boundary was set to the calculated mean arterial pressure.

Finally, the boundary conditions were set as follows:

1. At the entry of the aorta, the tangential velocity was set to 1 m/s, and the normal velocity was set to 0 m/s, as follows:

$$V_{t} \, = \, 1 {\text{m}}/{\text{s}},$$

$$V_{n} \, = \,0 {\text{m}}/{\text{s}},$$

where the subscripts *t* and *n* are the tangential and normal directions, respectively.

2. At the aorta exits, the normal and tangential outlet pressure were 0 mmHg and 93.09 mmHg, respectively (*p*_*t*2_ = 93.09 mmHg; *p*_*n*2_ = 0 mmHg).

The tangential outlet pressure of the LCA was 81.83 mmHg, and the normal pressure was 0 mmHg (*p*_*t*2_ = 81.83 mmHg; *p*_*n*2_ = 0 mmHg);

The tangential outlet pressure of the RCA was 92.71 mmHg, and the normal pressure was 0 mmHg (*p*_*t*2_ = 92.71 mmHg; *p*_*n*2_ = 0 mmHg).

The wall of the blood domain was assumed to have no slip, as follows: *v*_*n*_ = *v*_*t*_ = 0 m/s.

For the solid part, the boundary was constrained. A fluid load was applied to the inner surface of the vessel wall. The step end time was set to 1 s. The rest of the solid part was set to the default value.

Fluid–solid coupling follows the most basic conservation principle; therefore, the conservation of the fluid and solid stress, and the displacement and flow, should be satisfied at the fluid–solid coupling interface:

7 $$\sigma_{s} \cdot n_{s} = \sigma_{f} \cdot n_{f} ,$$

8 $$d_{s} = d_{f} ,$$

where *d* denotes the displacement vectors, *σ* denotes the stress tensors, *n* is the boundary normal, and subscripts *f* and *s* represent the fluids and solids, respectively.

### Numerical simulation

In this study, a one-way fluid–solid coupling simulation was conducted using ANSYS Workbench. The finite volume method (FVM) coupled with the finite element method (FEM) was used to solve the governing equations. The structural analysis of the vessel wall was carried out using ANSYS Mechanical. Additionally, ANSYS CFX (ANSYS CFX 19.0, Canonsburg, USA), which is a CFD software based on the finite element method, was used to carry out fluid analysis for the blood. The semi-implicit method for pressure-linked equations consistent (SIMPLEC) algorithm was used to couple the outflow velocity term, and all equations were solved using the separation solution method. The minimum number of iterations was set to one, and the maximum number of iterations was set to 100. The convergence criterion for the fluid portion was set to 1 × 10^−4^.

According to the Reynolds number, for Re < 2300, the blood flow was set to laminar; Re is defined as follows:

1 $$\text{Re} = \frac{\rho vd}{\mu },$$

where *v* and *ρ* are the fluid velocity and density, respectively. *d* is the characteristic length, and *μ* is the fluid viscosity.

### Statistical analysis

To eliminate patient specificity differences, multiple cases were statistically analyzed. The statistical analyses were performed using GraphPad Prism (GraphPad Software 8.0, CA, USA). Correlation analysis was carried out to evaluate the relationship amongst the area, flow rate, pressure, and WSS. The differences between the cross-sectional area of the inlet, volumetric flow, pressure, and WSS of the two groups were investigated by *t*-tests for two independent samples. The pressure and WSS values were collected using the five-point sampling method. If the correlation coefficient *r* was close to + 1, 0, and − 1, the results were positively correlated, negatively correlated, and not correlated, respectively. A correlation less than 0.5 was considered weak, whereas a correlation more than 0.8 was considered strong. The significance level was *P *< 0.05

## Data Availability

The datasets used and/or analyzed during the current study are available from the corresponding author on reasonable request.

## Abbreviations

LCA: The left coronary artery
RCA: The right coronary artery
AAOCA: Anomalous origin of the coronary artery
AORL: Anomalous origin of the right coronary artery from the left sinus
SCD: Sudden cardiac death
IVUS: Intravascular ultrasonography
CFD: Computational fluid dynamics
CT: Computed tomography
CTA: Computed tomography angiography
BMI: Body mass index
FVM: Finite volume method
FEM: Finite element method
SIMPLEC: Semi-implicit method for pressure-linked equations consistent
AAO: Ascending aorta
